# Supplementary material for: The Effect of Downsizing Packages of Energy-Dense, Nutrient-Poor Snacks and Drinks on Consumption, Intentions, and Perceptions—A Scoping Review
Source: Nutrients. 2021 Dec 21;14(1):9. doi: 10.3390/nu14010009 (PMC8746546; doi:10.3390/nu14010009)
Supplement: Supplementary file 1 [file nutrients-14-00009-s001.zip › nutrients-1502539-supplementary.pdf]

Figure S1: PRISMA-ScR Checklist

Preferred Reporting Items for Systematic reviews and Meta-Analyses extension for Scoping Reviews (PRISMA-ScR) Checklist

SECTION	ITEM	PRISMA-ScR CHECKLIST ITEM	REPORTED ON PAGE #
**TITLE**			
Title	1	Identify the report as a scoping review.	1
**ABSTRACT**			
Structured summary	2	Provide a structured summary that includes (as applicable): background, objectives, eligibility criteria, sources of evidence, charting methods, results, and conclusions that relate to the review questions and objectives.	1
**INTRODUCTION**			
Rationale	3	Describe the rationale for the review in the context of what is already known. Explain why the review questions/objectives lend themselves to a scoping review approach.	1-3
Objectives	4	Provide an explicit statement of the questions and objectives being addressed with reference to their key elements (e.g., population or participants, concepts, and context) or other relevant key elements used to conceptualize the review questions and/or objectives.	3
**METHODS**			
Protocol and registration	5	Indicate whether a review protocol exists; state if and where it can be accessed (e.g., a Web address); and if available, provide registration information, including the registration number.	3
Eligibility criteria	6	Specify characteristics of the sources of evidence used as eligibility criteria (e.g., years considered, language, and publication status), and provide a rationale.	3
Information sources*	7	Describe all information sources in the search (e.g., databases with dates of coverage and contact with authors to identify additional sources), as well as the date the most recent search was executed.	3-4
Search	8	Present the full electronic search strategy for at least 1 database, including any limits used, such that it could be repeated.	3-4
Selection of sources of evidence†	9	State the process for selecting sources of evidence (i.e., screening and eligibility) included in the scoping review.	4
Data charting process‡	10	Describe the methods of charting data from the included sources of evidence (e.g., calibrated forms or forms that have been tested by the team before their use, and whether data charting was done independently or in duplicate) and any processes for obtaining and confirming data from investigators.	4
Data items	11	List and define all variables for which data were sought and any assumptions and simplifications made.	4
Critical appraisal of individual sources of evidence§	12	If done, provide a rationale for conducting a critical appraisal of included sources of evidence; describe the methods used and how this information was used in any data synthesis (if appropriate).	4
Synthesis of results	13	Describe the methods of handling and summarizing the data that were charted.	4

SECTION	ITEM	PRISMA-ScR CHECKLIST ITEM	REPORTED ON PAGE #
**RESULTS**			
Selection of sources of evidence	14	Give numbers of sources of evidence screened, assessed for eligibility, and included in the review, with reasons for exclusions at each stage, ideally using a flow diagram.	5
Characteristics of sources of evidence	15	For each source of evidence, present characteristics for which data were charted and provide the citations.	5-6
Critical appraisal within sources of evidence	16	If done, present data on critical appraisal of included sources of evidence (see item 12).	6
Results of individual sources of evidence	17	For each included source of evidence, present the relevant data that were charted that relate to the review questions and objectives.	7-24
Synthesis of results	18	Summarize and/or present the charting results as they relate to the review questions and objectives.	25-27
**DISCUSSION**			
Summary of evidence	19	Summarize the main results (including an overview of concepts, themes, and types of evidence available), link to the review questions and objectives, and consider the relevance to key groups.	27-29
Limitations	20	Discuss the limitations of the scoping review process.	29
Conclusions	21	Provide a general interpretation of the results with respect to the review questions and objectives, as well as potential implications and/or next steps.	29-30
**FUNDING**			
Funding	22	Describe sources of funding for the included sources of evidence, as well as sources of funding for the scoping review. Describe the role of the funders of the scoping review.	30

JBI = Joanna Briggs Institute; PRISMA-ScR = Preferred Reporting Items for Systematic reviews and Meta-Analyses extension for Scoping Reviews.
* Where *sources of evidence* (see second footnote) are compiled from, such as bibliographic databases, social media platforms, and Web sites.
† A more inclusive/heterogeneous term used to account for the different types of evidence or data sources (e.g., quantitative and/or qualitative research, expert opinion, and policy documents) that may be eligible in a scoping review as opposed to only studies. This is not to be confused with *information sources* (see first footnote).
‡ The frameworks by Arksey and O'Malley (6) and Levac and colleagues (7) and the JBI guidance (4, 5) refer to the process of data extraction in a scoping review as data charting.
§ The process of systematically examining research evidence to assess its validity, results, and relevance before using it to inform a decision. This term is used for items 12 and 19 instead of "risk of bias" (which is more applicable to systematic reviews of interventions) to include and acknowledge the various sources of evidence that may be used in a scoping review (e.g., quantitative and/or qualitative research, expert opinion, and policy document).

From: Tricco AC, Lillie E, Zarin W, O'Brien KK, Colquhoun H, Levac D, et al. PRISMA Extension for Scoping Reviews (PRISMAScR): Checklist and Explanation. Ann Intern Med. 2018;169:467–473. doi: 10.7326/M18-0850.

Table S1: Search strategy

Database: Ovid MEDLINE, 1946 to 01 February, 2021
1 (energy dens* adj3 food*).tw. (1371)
2 snack*.tw. (8330)
3 (energy dens* adj3 low nutrient*).tw. (59)
4 (high energy adj3 nutrient* poor).tw. (10)
5 (non?recommend* adj3 food*).tw. (5)
6 (non?core* adj3 food*).tw. (21)
7 (high adj1 (fat* or sugar* food)).tw. (46262)
8 ((sugar* or sweet*) adj3 (beverage* or drink*)).tw. (6630)
9 ((soft or fizzy or carbonated) adj1 (beverage* or drink*)).tw. (4672)
10 SSB.tw. (5013)
11 (soda or pepsi or cola or coke or lemonade).tw. (8290)
12 (fruit adj1 drink*).tw. (436)
13 (food* adj3 (discretionary or junk or processed or package*)).tw. (6615)
14 convenien* food*.tw. (348)
15 packaged snack*.tw. (35)
16 inappropriate* food*.tw. (69)
17 confection#ry.tw. (951)
18 ((sweet* or savo#ry) adj2 food*).tw. (1483)
19 (candy or candies).tw. (1911)
20 (lolly* or lollies).tw. (47)
21 (sweets* or chocolate* or cake* or donut* or doughnut* or ice?cream* or dessert*).tw. (17739)
22 (biscuit* or cracker* or cookie* or waffle* or (snack adj2 bar*)).tw. (3847)
23 ((savo#ry or puff*) adj2 snack*).tw. (131)
24 (potato adj3 (chip* or crisp*)).tw. (752)
25 Snacks/ (1505)
26 Carbonated Beverages/ (2935)
27 1 or 2 or 3 or 4 or 5 or 6 or 7 or 8 or 9 or 10 or 11 or 12 or 13 or 14 or 15 or 16 or 17 or 18 or 19 or 20 or 21 or 22 or 23 or 24 or 25 or 26 (102869)
28 ((portion or packag* or container* or bottle* or can or cans or carton*) adj2 siz*).tw. (9481)
29 ((drink* or beverage* or snack* or confectionary) adj3 size*).tw. (311)
30 (food* adj3 packag*).tw. (4061)
31 (food* adj3 portion*).tw. (1062)
32 ((chocolate* or confection#ry or snack*) adj3 packag*).tw. (79)

33	Portion Size/ (529)
34	Food Packaging/ (4941)
35	28 or 29 or 30 or 31 or 32 or 33 or 34 (17978)
36	(Behavio?r* adj3 (consum* or customer* or individual)).tw. (15023)
37	(Behavio?r* adj3 (purchas* or select* or eat* or decision making or choice*)).tw. (25183)
38	(Food* adj3 (purchase or select* or eat* or consum* or intake* or choice* or preference*)).tw. (99857)
39	(satiet* or satiat*).tw. (12668)
40	Consumer Behavior/ (22007)
41	Health Behavior/ (51487)
42	Feeding Behavior/ (84321)
43	Food Preferences/ (14765)
44	Decision Making/ (97354)
45	36 or 37 or 38 or 39 or 40 or 41 or 42 or 43 or 44 (368896)
46	27 and 35 and 45 (1039)
47	limit 46 to english language (1011)

The full search strategy for other selected databases and grey literature will be provided upon request.

Table S2: Supplementary information of intervention studies comparing smaller versus larger single pack containing different total serving size of energy-dense, nutrient-poor snacks and drinks

First author, Year of publication, Country, Aim	Study sample	Study type	Experimental method	Setting	Potential moderators or mediators	Outcome measures	Results
Aerts, 2017 Study 1 (Belgium) To investigate the package size effect in children depending on food types.	96 (46 girls) Mean age 6.4±0.7 years	RCT[1]	Sweet popcorn (412 kcal/100 g) and salted popcorn (392 kcal/100 g); re-packed in plain popcorn cups (no brand slogan, logo, colour or nutrition information).<br>Between-subjects design; participants randomly assigned to the package condition.<br><br>60 g popcorn in a bucket<br>30 g popcorn in a bucket	Naturalistic<br>Watch TV while snacking in classroom for 1 hr	Food preference (sugared/ salted)<br>Age<br>Gender	Consumption	1. Children consumed significantly more (24 g/89%) from the larger pack than smaller pack (Mean small=27 g vs Mean large=51 g) for both sugared and salted popcorn, p<0.01.<br>2. The preference of popcorn was a moderator. The tendency to overconsume from the larger pack was higher) when served sugared popcorn (preferred) than salted popcorn, p<0.01.<br>3. Age and gender were not moderators.
Aerts, 2017 Study 2 (Belgium) To investigate the package size effect in children depending on food types.	55 (26 girls) Mean age 4.7±0.9 years	Crossover RCT	Ladyfinger cookies (400 kcal/100 g); re-packed in transparent container boxes (no brand slogan, logo, colour or nutrition information).<br>Within-subjects design; participants assigned to one of the four the package conditions in random order (four weekly experimental sessions in total).<br><br>48 g in a box<br>30 g in a box	Naturalistic<br>Watch TV while snacking in the classroom for 10 mins	Age<br>Gender	Consumption	1. Children consumed significantly more (7 g/30%) cookies from the larger pack than smaller pack (Mean small=26 g vs Mean large=33 g), p<0.01.<br>2. Age and gender were not moderators.

John,<br>2017 Study 2<br>(USA)<br>To test the effect of free refills on purchasing and consumption.	470 participants (211 females)<br>Mean age 33 years.	RCT	Sugary drinks (iced tea or lemonade); in clear plastic takeaway cups with lid and straw.<br><br>Between-subjects design; participants randomly assigned to package conditions.<br><br>One 680 ml cup<br>One 454 ml cup	Computer experiment<br>Complete an unrelated study in a 90 min session, participants were given \$0.4 to purchase a drink (large cup \$0.3, medium cup \$0.2)	None	Consumption<br>Likelihood of purchase	1. Participants who purchased a smaller-sized drink consumed significantly less than those who purchased a larger-sized drink (Mean medium=99.2±38.0 kcal vs Mean large 174.9±87.9 kcal).<br>2. The likelihood of purchase any drink between drink sizes did not differ, p=0.57.
John,<br>2017<br>Study 3a<br>(USA)<br>To test the effect of free refills on purchasing and consumption.	557 participants (261 females)<br>Mean age 32 years	RCT	Sugary drinks (iced tea or lemonade); in clear plastic takeaway cups with lid and straw.<br><br>Between-subjects design; participants randomly assigned to package conditions<br><br>One 567 ml cup<br>One 454 ml cup	Computer experiment, participants completed an unrelated study in a 90-min session, participants were given \$0.4 to purchase a drink (large cup \$0.3, medium cup \$0.2)	None	Consumption<br>Likelihood of purchase	1. Participants who purchased a smaller-sized drink consumed significantly less than those who purchased larger-sized drink, $p<0.01$.<br>2. The likelihood of purchase any drink between drink sizes did not differ, p=0.26.
Marchiori 2012<br>(Belgium)<br>To examine whether greater food intake would<br>be observed with larger containers despite holding food portion constant.	88 under-graduate students (62 females)<br>Mean age 20.1±2.1 years	RCT	M&M's; pre-packed by experimenters in aluminium boxes.<br>Between-subjects design; participants randomly assigned to the package condition.<br><br>600 g in a box<br>200 g in a box	Face-to-face experiment<br>Watch a 22-min TV show in individual cubicles in the laboratory while snacking	Age<br>Food preference<br>Weight	Consumption	1. Participants consumed significantly less (30 g/150 kcal (50%)) from the smaller (200 g) box than larger (600 g) box (Mean small=30 g/155 kcal vs Mean large-fully-filled=60 g/305 kcal, respectively), $p<0.002$.<br>2. Age, food preference and weight were not moderators.
Rolls, 2004<br>(USA)<br>To test the effect of the portion	60 (34 female)<br>Mean age 22.9 years	Crossover RCT	Potato chips; re-packed in opaque bags with five package size conditions.<br>Within-subjects design; participants assigned to one of the five the package conditions in random order.	Face-to-face experiment<br>15-min snack session, subsequent dinner in individual	Gender<br>Age<br>Dietary restraint status<br>Weight	Consumption	1. The overall reduction in consumption from the largest (170 g) to smallest (28 g) packages (females 184 kcal less, males 311 kcal less) was significant when the package size was incrementally reduced from 170 g to 28 g.

size of a snack food on intake by providing packages of potato chips in five sizes.			Larger bags: 85 g, 128 g, 170 g<br>Smaller bags: 28 g, 42 g	cubicles in the laboratory.<br>Five visits on five separate days			2. This effect was more prominent for males than females, p<0.01.<br>3. When considering snack and dinner intake together, the total intake increased as the snack package size increased, significant for both genders.<br>4. Age, dietary restraint and weight were not moderators.
Versluis, 2016 Study 2 (Netherland)<br>To investigate if providing a diet prime is effective for reducing the magnitude of the pack size effect.	224 university students (92 females)<br>Mean age 21±1.6 years	RCT	M&M's.<br>Between-subjects design; participants randomly assigned to the package condition<br><br>400 g bag<br>200 g bag	Face-to-face experiment<br>Watch a 16-min movie clips and commercials on individual computer while snacking in individual cubicles	Diet prime (commercials) [3]<br>Dietary restraint status<br>Food preference<br>Gender<br>Weight	Consumption	1. No significant effect of package size on consumption was found (p=0.41).<br>2. When exposing to a diet prime prior to eating, restrained eaters consumed significantly less from larger pack, but not from smaller pack. Exposing to a diet prime prior to eating did not influence consumption in unrestrained eaters.<br>3. Dietary restraint, food preference, gender and weight were not moderators.
Wansink, 2001 (USA)<br>To examine how container sizes interact with palatability and in turn, impact food consumption.	151 moviegoers (66 females)<br>Age range 11-89 years	RCT	Popcorn.<br>Between-subjects design; participants randomly assigned to the package condition.<br><br>240 g in a bucket<br>120 g in a bucket	Naturalistic setting<br>Watch movie in movie theatre while snacking, followed by a questionnaire about perceived taste and healthfulness of popcorn	Food preference (perceived taste)	Consumption<br>Perception (healthiness)	1.Participants consumed significantly less (33 g/35%) from the smaller pack than larger pack (240 g to 120 g) (Mean small=61 g vs Mean large=94 g), p<0.01.<br>2.Food preference was a moderator. This effect was more prominent in participants who rated the taste as favourable than those who rated the taste as unfavourable, p<0.05.<br>3.Participants tended to pay more attention to monitor their intake when eating from the smaller pack, p<0.01.<br>4. Participants tended to perceive popcorn in smaller packs to be healthier than the larger pack, p<0.01
Wansink, 2005 (USA)<br>To investigate whether environmental cues such as packaging and container size can	158 moviegoers (67 females)<br>Mean age 28.7 years	RCT	Popcorn<br>Between-subjects design; participants randomly assigned to the package condition.<br><br>240 g in a bucket<br>120 g in a bucket	Naturalistic setting<br>Watch movie in movie theatre while snacking, followed by completing a one-page questionnaire about their perception of the popcorn	Food preference (fresh/stale)	Consumption	1. Participants consumed significantly less (20 g/28%) from the smaller pack than larger pack (240 g to 120 g).<br>2. Food preference was a moderator. This effect was more prominent for the fresh popcorn (Mean small=59 g vs Mean large=86 g) than for the stale popcorn (Mean small=38 g vs Mean large=51 g), all ps<0.01.

increase intake of foods that are less palatable.							
Clarke, 2020 (UK) To estimate the impact of plate size and shape on amount of food self-served, and glass and bottle size on amount of wine self-poured.	140 adult regular wine drinkers (96 females). Mean age 41 years	Crossover RCT	Wine, in original branded bottles.<br><br>Within-subjects design; participants assigned to each package condition in random orders.<br><br>750 ml bottle<br>500 ml bottle	Face-to-face experiment Participants indicated typical serving size for one meal by pouring wine from bottles to glasses; wine bottle size and glass size were both manipulated (6 conditions in total)	Gender	Intention to consume	1. No effect of wine bottle size on intention to consume was found, p=0.20.<br>However, there was an overall effect of wine glass size, regardless of bottle size.<br>2. No interaction between wine bottle size and wine glass size was observed, p=0.18.<br>3. Gender was not a moderator.
Versluis, 2015 Study 1 (Netherland) To test whether providing a serving size recommenda-tion would reduce the influence of the pack size on consumption.	317 (159 females) Mean age 44±12 years	RCT	Milk chocolate; snack food images were similar to the original manufacturer package (front side). Between-subjects design; participants randomly assigned to the package condition.<br><br>180 g bar<br>75 g bar	Computer-based experiment Picture of chocolate displayed on computers, participants indicated intended consumption and additional questionnaires on individual computers	Gender Serving size recommenda-tion labelling (pictorial)[4]	Intention to consume	1. Participants intended to consume significantly less (11g/56 kcal (22%)) from the smaller pack than larger pack (Mean small=40 g vs Mean large=51 g), p<0.001.<br>2.. Gender was a moderator. This effect was only significant among males, p<0.01.<br>3. Serving size recommendation labelling was not a moderator.

Versluis, 2015 Study 2 (Netherland) To test whether providing a serving size recommenda-tion would reduce the influence of the pack size on consumption.	324 (154 females) Mean age 38±11 years	RCT	Milk chocolate, M&M's, savory crackers; snack food images were similar to the original manufacturer package (front side).<br>Between-subjects design; participants randomly assigned to the package condition<br><br>Milk chocolate 180 g vs 75 g<br>M&M's: 400 g vs 165 g<br>Crackers: 120 g vs 60 g	Computer-based experiment Picture of chocolate displayed on computers, participants indicated intended consumption of all snacks and additional questionnaires on individual computers	Gender Serving size recommenda-tion labelling (pictorial)	Intention to consume	1. Participants intended to consume significantly less (22 g/27%) from the smaller pack than larger pack (Mean small=59 g vs Mean large=81 g) for each snack, p=0.01.<br>2. This effect was significant for both genders, but it was more prominent for males than for females, p=0.02.<br>3. The pictorial serving size recommendation labelling resulted in lower intention to overconsume when package size was large but not when was small.
Versluis, 2016 Study 1 (Netherland) To investigate if providing a diet prime is effective for reducing the magnitude of the pack size effect.	477 (244 females) Mean age 40±11 years	RCT	Milk chocolate, M&M's, potato chips; snack food images were similar to the original manufacturer package (front side).<br>Between-subjects design; participants randomly assigned to the package condition<br><br>Milk chocolate: 180 g vs 75 g<br>M&M's: 400 g vs 165 g<br>Potato chips: 300 g vs 120 g	Computer-based experiment Read magazines (non-diet or diet prime) before picture of snacks displayed on computer, then indicated intended consumption of each snack on individual computer	Diet prime (health magazines)[5]	Intention to consume	1.Diet prime was a moderator.<br>Participants who were exposed to non-diet prime (travel magazine, as the control group) prior to eating had significantly lower intention to consume from smaller than larger pack, p<0.01.<br>2. Exposing to diet prime prior to eating diminished this effect by reducing the tendency to overconsume from the larger pack, no difference in intention to consume between the smaller and larger pack was found, p=0.97.
Wansink, 1996 Study 4 (USA) To investigate whether larger package sizes encourage greater use than smaller package size, and whether the unit size mediates	184 (all females) 39 participants completed the follow-up questionnair e	Quasi[2]	M&M's; package modifications were made.<br>Between-subjects design; provided 3 types of food (oil, spaghetti, M&M's).<br><br>Large bag: 342 chocolates<br>Medium bag: 228 chocolates<br>Small bag: 114 chocolates	Computer-based experiment Indicate intention to consume by scooping M&M's into a bowl. Perceived cost of the use of M&M's was also assessed	None	Intention to consume Perception of snack unit prices	1. Participants intended to consume significantly more (40 g/63%) from the medium pack than small pack (Mean small=63 pieces vs Mean medium=103 pieces), p<0.05.<br>2. Participants intended to consume significantly more (59 g/94%) from the large pack than small pack (Mean small=63 pieces vs Mean large=122 pieces), p<0.05.<br>3. No significant difference in intention to consume was found between medium- and large-sized packs.<br>4. Unit price was a moderator. Participants perceived the unit prices to be higher when package sizes became smaller (that is, perceived unit cost was the highest for the small pack).

this relationship.							
Huyghe, 2013 (Belgium)<br>To investigate whether consumers prefer bonus packs as opposed to price discounts for indulgent foods.	235 (157 females)<br>Mean age 32.4 ± 13.8 years	Quasi	Chocolate cookie, muffin, chocolates, chocolate bar.<br>Between-subjects design; participants assigned to four of the 32 conditions (in which the price or package size was manipulated across participants in each trial).<br><br>40 g, 80 g, 120 g, 160 g, 200 g, 240 g, 280 g, 320 g	Computer-based experiment<br>Complete online questionnaire that consisted of four trials, each trial presented one snack food and one healthy food option	Gender	Intention to purchase	1. No effect of snack package size on intention to purchase was found.<br>The price of snacks had a stronger effect on intention to purchase than the package size had. When the unit price increased to the same extent (by reducing the package size and price unchanged or increasing the price and package size unchanged), participants were more responsive to the price increase than the package reduction.<br>2. Gender was not a moderator.

[1.] RCT: randomised-controlled trials.

[2.] Quasi: quasi-experimental studies.

[3.] Diet prime: diet-related commercials with messages focused on resisting temptation of foods (for example, dieting, setting and reaching goals, weight loss plan); Non-diet prime (control group): non-diet-related commercials, no message related to dieting, food, or exercise.

[4.] Pictorial serving size recommendation labelling: using picture of snack food in nutrition labelling (e.g. a picture of four pieces of chocolates as the recommended serving size), which is different from non-pictorial labelling that uses text.

[5.] Diet prime: health magazine with messages related to weight loss, diets, and fitness; Non-diet prime (control group): travel magazine, no message related to dieting, food, or exercise.

Table S3: Supplementary information of intervention studies comparing smaller multipacks versus larger pack(s) containing same total serving size of energy-dense, nutrient-poor snacks and drinks

First author, Year of publication, Country, Aim	Study sample	Study type	Experimental method	Setting	Potential moderators or mediators	Outcome measures	Results
Argo, 2012 Study 2 (Canada)<br>To examine whether people low in appearance self-esteem are particularly sensitive to external control properties.	207 under-graduate students (123 females)	Quasi[1]	Candy-coated chocolates; the weight of small/large packaging units were not reported.<br>Between-subjects design.<br><br>Two larger packs<br>Eight smaller packs	Face-to-face experiment<br>Complete a survey on snacks (chocolates) while snacking in laboratory setting	Package design (transparent/ opaque)<br>Appearance self-esteem (ASE)[3]	Consumption	1. Participants consumed significantly more from the smaller multipacks than larger packs, $p<0.01$. This was fully contributed by those who had low-ASE.<br>2. Participants with low-ASE consumed significantly more (29 g/193%) from the smaller multipacks than larger packs (Mean small= 44 g vs Mean large 15 g), $p<0.01$. No effect was found among those with high-ASE.<br>3. Package design was a moderator. When packaging was transparent (vs opaque), participants consumed significantly more (42 g/100%) from the smaller multipacks than larger packs, $p<0.01$.<br>4. Gender was a moderator
Bui, 2017 Study 3 (USA)<br>To examine how perceived food healthfulness and package partitioning interact to impact intended and actual consumption.	67 under-graduate students (35 females)<br>Mean age 27 years	Quasi	Bite-sized chocolate chip cookies; packages were re-designed to look similar to the original manufacturer package.<br>Between-subjects design; participants randomly assigned to one of the four conditions (perceived healthy food - cookies versus perceived unhealthy food - granolas in smaller or larger packs).<br><br>One larger bag (16 pieces each bag)<br>Four smaller bags (4 pieces each bag)	Face-to-face experiment<br>Read study instruction while snacking for 5 mins in laboratory setting	Gender	Consumption	1. No significant effect of package size on consumption was found, $p>0.5$.<br>2. Gender was not a moderator.
Codling, 2020 (UK)<br>To assess the impact of purchasing wine in 50 cl bottles compared with 75	166 households (adult household members were regular	Cross-over RCT[2]	Wine; given in original packages; total serving size was consistent between package conditions.<br>Within-subjects (households) design; participants assigned to one of the two package conditions in random order,	Free-living environment<br>Participants purchased wine from retailers (for example, 2-week 500ml	The order of receiving each package condition	Consumption	1. Participants (households) consumed significantly less (173 ml/4%) wine in 14 days from the 500ml bottles than 750ml bottles (Mean small=4206 ml vs Mean large=4379 ml), $p<0.01$.<br>2. Participants had a significantly lower rate of consumption (6%) from the 500ml bottles than 750ml bottles, $p<0.05$

cl bottles on the amount of wine consumed at home.	wine drinkers-) Mean age 31 years		750ml bottles<br>500ml bottles	bottles; 3-week washout period; another 2-week 750ml bottles)			3. The order of receiving each package size condition was not a moderator.
Do Vale 2008 Study 2 (Netherland)<br>To examine actual consumption behaviour with different package formats.	140 under-graduate students (59 females)	RCT	Potato chips.<br>Between-subjects design; participants randomly assigned to package conditions.<br><br>Two 200 g packs (total serving size 400 g)<br>Nine 45 g packs (total serving size 405 g)	Face-to-face experiment<br>Complete advertisement evaluation while snacking for 20 mins in laboratory setting	Self-regulatory concern[4]	Consumption	1. No significant effect of package size on consumption was found (Mean small=49 g vs Mean large=50 g), p=0.2.<br>2. Participants were significantly more likely to open smaller multipacks than larger packs (Mean small=66.2% vs Mean large=38.9%), p<0.01; and smaller multipacks required shorter deliberation of being opened.<br>3. The activation of self-regulatory concern led to lower intake from larger packs (but not from smaller multipacks), which diminished the package size effect.
Haire, 2014 (USA)<br>To examine whether weight and restraint status influence the relationship between package size and consumption.	64 university students (30 females)<br>Mean age 23.7 years	RCT	Mini-pretzels (energy density of 3.7 kcal/g); in original manufacturer packages.<br>Between-subjects design; participants randomly assigned to package conditions.<br><br>Two 283 g packs<br>Twenty-two 26 g packs	Free-living experiment<br>Participants consumed snacks in real life for 4 days	Weight (22.2 kg/m^2 in normal weight group; 29.8 kg/m^2 in overweight group)<br>Dietary restraint status	Consumption	1. Overweight or obese participants consumed significantly less (97 g/361 kcal (48%)) from the smaller multipacks than larger packs. (Mean small=107 g vs Mean large=204 g), p=0.01.<br>2. No significant effect of package size was found among normal weight participants.<br>3. Dietary restraint was not a moderator.
Holden, 2015 Study 1 (Australia)<br>To test the effect of breaking a portion into multiple smaller partitions smaller on consumption and the role of dietary restraint.	108 university students (58 females)	Quasi	M&M's; re-packed in clear plastic bags.<br>Between-subjects design.<br><br>One 200 g pack<br>Four 50 g packs	Face-to-face experiment<br>Complete a 10-min questionnaire while snacking in laboratory setting	Manipulated diet consciousness[5]<br>Measured diet consciousness[6]	Consumption	1. Participants consumed significantly more (10 g/67%) from the smaller multipacks than larger pack (Mean small=25 g vs Mean large=15 g), p=0.004. This effect was mainly contributed by those with activated diet-consciousness concern.<br>2. Participants consumed significantly more (29 g/161%) from the smaller multipacks than larger pack when their diet-consciousness concern was activated (Mean small=47 g vs Mean large=18 g), p<0.01; no effect was found when diet consciousness was not activated.<br>3. No significant effect of package size was found among those with higher diet-consciousness. Participants with higher diet-consciousness consumed slightly more (9 g/69%) from the smaller multipacks than larger pack (Mean small=22 g vs Mean large=13g, not statistically significant).

Holden, 2015 Study 2<br>(Australia)<br>To test the effect of breaking a portion into multiple smaller partitions smaller on consumption and the role of dietary restraint.	114 university students (64 females)	Quasi	M&M's; re-packed in clear plastic bags.<br>Between-subjects design<br><br>One 200 g pack<br>Four 50 g packs	Face-to-face experiment<br>Complete a 10-min questionnaire while snacking in laboratory setting	Diet consciousness [7]<br>Diet prime (food-focused)[8]	Consumption	1. No significant effect of package size on consumption was found (diet consciousness was activated in all participants).<br>2. Food-focused diet-prime was a moderator, p=0.01. Participants' tendency to overconsume from the smaller multipacks disappeared when a food-focused diet prime was provided. (Mean small=20 g vs Mean large=25 g, but not statistically significant).
John,<br>2017 Study 1<br>(USA)<br>To test the impact of bundling on purchasing and consumption, and whether its effect depends on service style.	362 drink purchasers (out of 623 partici-pants). Mean age 24 years.	RCT	Sugary drinks (iced tea or lemonade); in clear plastic takeaway cups with lid and straw.<br><br>Between-subjects design; participants randomly assigned to package conditions.<br>Package size options were randomised and had two options (large and medium; or small and medium)<br><br>Large: one 680 ml cup<br>Small: two 340 ml cups<br>Medium (control): one 454 ml cup	Computer experiment<br>Complete an unrelated study in a 90-min session, participants were given $0.4 to purchase a drink (large cup $0.3, two small cups $0.3, medium cup $0.2)	None	Consumption<br>Likelihood of purchase	1. No significant effect of package size on consumption was found. Participants consumed slightly less from multiple smaller cups than one larger cup (Mean small=115.7±49.8 kcal vs Mean large=122.6±47.9 kcal, not significant), p=0.23.<br>2. Among participants who chose to purchase a drink, those in the two 340 ml cups condition had a significant higher likelihood of purchase compared to those in the one 680ml cup condition (39.5% participants purchased a 680ml cup while 25.6% participants purchased two 340ml cups), p<0.01.
Kerameas, 2015 Study 1<br>(Australia)<br>To test the segmentation effect that people eat less when a unit of food is separated into smaller subunits.	87 female undergrad-duate students<br>Mean age 20 years<br>Mean BMI 21.8 kg/m2	Quasi	Cookies; in sealed, clear cellophane bags. Each cookie was individually wrapped.<br>Between-subjects design. Four package conditions – the total serving sizes were either 30 g or 90 g.<br><br>Smaller total serving size conditions (30 g):<br>One 30 g cookie in one larger bag<br>Three 10 g cookies in three smaller	Face-to-face experiment<br>Complete a 10-min survey while snacking in laboratory setting; additional cookies were available	Perceived norm of appropriate intake[9]	Consumption	1. Participants consumed significantly less from the smaller multipacks than a larger pack (Mean small package=53 g vs Mean large package=70 g), p=0.01.<br>2. Although additional cookies were available, participants in the 30 g total serving size conditions consumed significantly less compared to those in the 90 g total serving size conditions (Mean 30 g serving=46 g, Mean 90 g serving=77 g), p<0.01.<br>3. The perceived norm of appropriate intake was a mediator. Participants reported a lower perceived norm of appropriate intake when served the multiple smaller packages than a

			bags<br><br>Larger total serving size conditions (90 g):<br>One 90 g cookie in one larger<br>Three 30 g cookies in three smaller bags				larger package (Mean small=53.8±33.9 g vs Mean large=76.8±27.7 g), p<0.01.
Mantzari, 2017 (UK)<br>To explore the feasibility and acceptability of conducting a randomised controlled trial to assess the impact of different bottle sizes on beverage consumption at home.	16 household representa-tives (12 females), purchased at least 2 L of regular cola drinks per week<br>Mean age 33±6.6 years	Cross-over RCT	Cola, in original manufacturer bottles; total serving size was consistent between package conditions.<br>Within-subjects (households) design; participants assigned to one of the four package conditions in random order.<br><br>1500 ml, 1000 ml, 500 ml, 250 ml bottles	Free-living experiment<br>Participants consumed cola in real life for 4 weeks (each bottle size per week), followed by a semi-structured interview	None	Consumption	1. No powered significance testing was undertaken as it was a feasibility study.<br>The average weekly consumption of 250ml, 500ml, 1000 ml, and 1500 ml bottle size per household was 7878±3861 ml, 8595±3559 ml, 8331±3963 ml, 8010±3977 ml, respectively.
Mantzari, 2020 (UK)<br>To explore the feasibility and acceptability of conducting a large-scale randomised study to assess the impact of bottle size on in-home wine consumption.	16 households, regular wine drinkers.<br>Mean number of adult drinkers in each household was 1.7<br>Mean age 40±2.7 years	Cross-over RCT	Wine, in original bottles; total serving size according to regular consumption amount, consistent between package conditions.<br>Within-subjects (households) design; participants assigned to one of the two package conditions in random order<br><br>750 ml, 375 ml bottles	Free-living experiment<br>Participants consumed wine in real life (each bottle size every 2 weeks); wine was delivered to households fortnightly	The order of receiving each package condition	Consumption	**1.** Household consumption in 2 weeks was 8.4 ml lower when receiving smaller bottles than larger bottles (Mean small=3378±1719 ml vs Mean large=3385±1699 ml).<br>2. The order of receiving each package condition could be a potential moderator (but no powered significance testing was undertaken as it was a feasibility study). In four weeks, households receiving smaller bottles first consumed 1020 ml less wine overall than those receiving the larger bottles first (Mean small first=6316±3294 ml vs Mean large first=7336±3736 ml).
Raynor, 2007 (USA)<br>To examine the	24 healthy, non-obese adults (12 female), regular	RCT	A snack box with 4 types of snacks; in original manufacturer packages.<br>Potato chips, crackers, Oreo miniature cookies; M&M's.	Free-living experiment<br>Participants consumed	Gender<br>Weight	Consumption	1. The total serving size had a significant effect on consumption, regardless of package size. Smaller total serving size resulted in a significantly lower snack consumption in 3 days, p<0.01.

independent effects of package unit size and amount of food on intake.	snack consumer<br>Mean age 20±1.6 years		Between-subjects design; participants randomly assigned to package conditions.<br><br>Larger packs were at least 5 times the size of the smaller packs 140-263 g (total serving size 8748 or 4374 kcal)<br>Smaller packs 28-48 g (total serving size 8820 or 4320 kcal)	snacks in real life for 3 days			2. No significant effect of package size on consumption was found (Mean small=4027 kcal vs Mean large=3783 kcal), p>0.1.<br>3. Gender and weight were not moderators.
Roose,<br>2017 Study 2<br>(Belgium)<br>To examine the effect of food granularity on consumption in a controlled setting.	188 university students (88 females)<br>Mean age 22 years	RCT	Brownies; in paper bags; total serving size was 100 g.<br>Between-subjects design, participants randomly assigned to package conditions.<br><br>One larger bag of 6 brownies<br>Three smaller bags (2 brownies per bag)	Face-to-face-experiment<br>Watch movies while snacking for 10 mins	Self-control conflict[10]<br>Dietary restraint status[11]	Consumption	1. Participants consumed significantly more (13 g/30%) from the smaller multipacks than a larger pack (Mean small 54.6±4.0 g vs Mean large=42.0±4.0 g), p=0.03.<br>This effect was significant among restrained eaters only, p=0.02. No effect was found among unrestrained eaters.<br>2. The self-control conflict was a mediator. Participants experienced less self-control conflict when consuming from smaller packages than larger pack.
Scott, 2008 Study 2<br>(USA)<br>To examine the moderating role of dietary restraint on the amount of food consumed from small food in small packages versus large food in<br>large packages.	343 university students	Quasi	M&M's; re-packed in clear plastic bags.<br>Between-subjects design.<br><br>One 200 kcal pack of regular-sized M&M's<br>Four 50 kcal packs of mini M&M's	Face-to-face experiment<br>Complete a questionnaire unrelated to the experiment while snacking in experimental room for 40 mins	Dietary restraint status	Consumption<br>Perception (energy content)	1. Participants consumed significantly less from the smaller multipacks with mini M&M's than a larger pack with regular-sized M&M's, p<0.05.<br>2. Unrestrained eaters consumed significantly less (48 kcal/38%) from the smaller multipacks with mini M&M's than larger pack with regular-sized M&M's.<br>3. Restrained eaters tended to eat more (12 kcal/12%) from smaller multipacks with mini M&M's than a larger pack with regular M&M's (but not statistically significant).<br>4. Participants perceived the energy content of smaller multipacks with mini M&M's to be significantly greater than a larger pack with regular M&M's, and perceived mini M&M's in smaller multipacks to be more like diet food than regular-sized M&M's in a larger pack.
Scott, 2008 Study 3<br>(USA)<br>To examine the moderating role of dietary restraint on the amount of food	96 under-graduate marketing students	Quasi	Cookies; re-packed in clear plastic bags.<br>Between-subjects design.<br><br>One 240 kcal pack of regular-sized cookies (4 regular cookies per pack)	Face-to-face experiment<br>Complete a questionnaire unrelated to the experiment	Dietary restraint status	Consumption<br>Perception (prediction of consumption)	1. No significant package size effect (mini cookies in smaller multipacks vs regular-sized cookies in larger pack) was found.<br>2. Participants predicted that they would consume less from smaller multipacks with mini cookies than a larger pack with regular-sized cookies, p<0.01.

consumed from small food in small packages versus large food in large packages.			Four 60 kcal packs of mini cookies (2 mini cookies per pack)	while snacking in experimental room for 40 mins			3. Dietary restraint was not a moderator.
Scott, 2008 Study 4 (USA)<br>To examine the moderating role of dietary restraint on the amount of food consumed from small food in small packages versus large food in large packages.	393 under-graduate business students	Quasi	M&M's, re-packed in clear plastic bags.<br>Between-subjects design<br>One 200 kcal pack of regular-sized M&M's<br>Four 50 kcal packs of mini M&M's	Face-to-face experiment<br>Complete a questionnaire unrelated to the experiment while snacking in experimental room for 40 mins	Dietary restraint status<br>Diet prime (food-focus)[12]	Consumption<br>Perception (perceived caloric content)	1. Participants consumed significantly less from smaller multipacks with mini M&M's than a larger pack with regular-sized M&M's, p<0.01.<br>2. Participants perceived smaller multipacks with mini M&M's to be significantly more like diet food and contain higher energy than a larger pack with regular M&M's (Mean small=321 kcal vs Mean large=219 kcal), ps <0.01.<br>3. Food focus was a moderator for restrained eaters but not for unrestrained eaters. When considering M&M's as non-food projects (non-food focus), restrained eaters consumed significantly less (60 kcal/59%) from smaller multipacks than a larger pack (Mean small=42 kcal vs Mean large=102 kcal), p<0.001.<br>In control group when there was no food focus, restrained eaters consumed significantly more (33 kcal/43%) from smaller multipacks than a larger pack (Mean small=109 kcal vs Mean large=76 kcal), p<0.05.<br>4. Food focus had no effect in unrestrained eaters. They consumed significantly less (45 kcal/33%) from smaller multipacks than a larger pack.
Stroebele, 2009 (USA)<br>To determine whether the portion-controlled packages result in less consumption as compared to larger packages when the amount of food provided was held constant.	59 (41 females)<br>Mean age 37.3 ± 12 years<br>Mean BMI 27.7 ± 3.9 kg/m2	Cross-over RCT	10 types of snacks, all in original manufacturer packages:<br>Cool goldfish, Baked Ritz Chips, Snyder's Pretzels, Cheese Nips, Multigrain Wheat Thins, Cinnamon Teddy Grahams and Lorna Doone shortbread cookies.<br>Within-subjects design; participants assigned to one of the two package conditions in random order.<br><br>Four larger packs (187-360 g per pack)	Free-living experiment<br>Participants consumed snacks for 2 weeks in real life (one package condition per week)	The order of receiving each package size condition	Consumption	1. On a weekly basis, participants consumed significantly less (187 g/32%) from smaller multipacks than larger packs (Mean small=395 g vs Mean large=581 g), p<0.001.<br>2. Participants who received smaller multipacks first (in week 1) consumed significantly less snacks (28%) from larger packs later (in week 2), compared to those who received larger packs first and smaller multipacks later, p=0.05.

			Accordingly number of smaller packs (19-26 g per pack) to keep the total serving size consistent				
Van Kleef, 2014 Study 3 (Netherland)<br>To examine whether the unit size of food impacts intake as it influences perceptions of impulsiveness and appropriateness.	165 university students (104 females)<br>Mean age 21±2.4 years	RCT	Mars chocolate bars, in original manufacturer package.<br>Between-subjects design; participants randomly assigned to the package condition.<br><br>Three 51 g chocolate bars (228 kcal each bar, total energy 684 kcal)<br>Fifteen 10 g chocolate bars (45 kcal each bar, total energy 675 kcal)	Face-to-face experiment<br>Complete a 10-min task on non-food-commercial, do the evaluation while snacking in laboratory setting.	Perception of impulsiveness [13]<br>Weight	Consumption<br>Perception (satiety)	1. Participants consumed significantly less (51 kcal/23%) from smaller multipacks than larger packs (Mean small=171 kcal vs Mean large=222 kcal), p<0.01.<br>2. The perceptions of impulsiveness was a mediator, p=0.02. Participants counteracted the feelings of impulsiveness by eating less from smaller multipacks.<br>3. Larger packs were perceived to be more satiating than smaller multipacks, p=0.05.<br>4. Weight was not a moderator.
Wansink, 2011 (USA)<br>To assess whether 100-calorie sub-packaging reduce intake and enable individuals to accurately track intake.	37 university students (15 females)<br>Mean age 20.3±1.1 years<br>Mean BMI 23.8±3.9 kg/m2	RCT	Crackers.<br>Between-subjects design; participants randomly assigned to the package condition.<br><br>One 400 kcal pack<br>Four 100 kcal packs	Face-to-face experiment<br>Watch a 22-min TV comedy while snacking in laboratory setting	Weight	Consumption	1. Participants consumed significantly less (75 kcal/25%) from smaller multipacks than a larger pack (Mean small=223 kcal vs Mean large=298 kcal), p=0.006.<br>2. This effect was only significant in overweight participants, they consumed significantly less (208 kcal/54%) from smaller multipacks than a larger pack, p=0.001.<br>3. No significant effect of package size on consumption was found in normal-weight participants, p>0.99.<br>4. No significant effect of package size on the feeling of fullness after consumption was found, p=0.10. However, participants consuming smaller multipacks claimed that they would eat less for their next meal, p=0.01.

Bui, 2017 Study 1 (USA)<br>To examine how perceived food healthfulness and package partitioning interact to impact intended and actual consumption.	77 postgra-duate students (44 females)<br>Mean age 31 years	Quasi	Bite-sized chocolate chip cookies; packages were re-designed to look similar to the original manufacturer package.<br>Between-subjects design; participants were assigned to package conditions<br><br>One larger pack (16 pieces of cookies each bag)<br>Four smaller packs (4 pieces of cookies each bag)	Computer-based experiment<br>Complete a survey on intention to consume for displayed snacks (cookies) in laboratory setting	None	Intention to consume	No significant effect of package size on intended consumption was found for cookies (which were perceived as an 'unhealthy food'), p>0.5.
Bui, 2017 Study 2 (USA)<br>To examine how perceived food healthfulness and package partitioning interact to impact intended and actual consumption.	171 (103 females)<br>Mean age 38 years	Quasi	Bite-sized chocolate chip cookies, packages were re-designed to look similar to the original manufacturer package.<br>Between-subjects design<br><br>Two larger packs (8 pieces each bag)<br>Four smaller packs (4 pieces each bag)	Computer-based experiment<br>Complete a survey on intention to consume for displayed snacks (cookies) in laboratory setting	None	Intention to consume	No significant effect of package size on intended consumption was found for cookies (which were perceived as an 'unhealthy food'), p>0.4.
Scott, 2008 Study 3- follow-up (USA)<br>To examine the moderating role of dietary restraint on the amount of food consumed from small food in small packages versus large food in large packages.	201 under-graduate business students	Quasi	M&M's; re-packed in clear plastic bags.<br>Between-subjects design.<br><br>One 200 kcal pack of regular-sized M&M's<br>Four 50 kcal packs of mini M&M's	Face-to-face experiment<br>Observe M&M's and complete a questionnaire on intention to consume and stress level	Dietary restraint status	Intention to consume	1. Participants (both restrained and unrestrained eaters) intended to eat less (23%) from smaller multipacks with mini M&M's than larger packs with regular-sized M&M's (Mean small=41% vs Mean large=64%), p<0.01.<br>2.Dietary restraint status was not a moderator.<br>However, restrained eaters perceived that considering the consumption of mini M&M's from smaller multipacks to be significantly more stressful than regular-sized M&M's from a larger pack, p<0.05.<br>This effect was not observed in unrestrained eaters, p>0.5.
Mantzari, 2018 (UK)<br>To explore the experiences of	16 household representa-tives (12 females),	Qualitati ve study	Cola, in original manufacturer bottles; total serving size according to regular consumption amount, consistent between package conditions.	Free-living experiment<br>Participants consumed cola in real life for 4	None	Perception of previous consumption (rate and amount)	1. Participants believed that their consumption rate and amount was higher with the smallest (250mL) bottle size due to the perception of more convenient, reduced awareness of the amount consumed, harder for consumption monitoring, and insufficient quantity in each bottle.

consuming cola from smaller compared with larger bottles, to inform intervention strategies.	purchased at least 2 L of regular cola drinks per week<br>Mean age 33±6.6 years		Within-subjects (households) design; participants assigned to one of the four package conditions in random order.<br><br>1500 ml, 1000 ml, 500 ml, 250 ml bottles	weeks (each bottle size per week), followed by a semi-structured interview			Small bottle sizes might increase the frequency of consumption occasions and lead to consumption of numerous bottles in succession.
Scott, 2008 Study 1 (USA)<br>To examine the moderating role of dietary restraint on the amount of food consumed from small food in small packages versus large food in large packages.	385 under-graduate business students	Quasi	M&M's; re-packed in clear plastic bags without nutrition information. Between-subjects design.<br><br>One 200 kcal pack regular-sized M&M's<br>One 200kcal pack mini M&M's<br>Four 50kcal packs regular-sized M&M's<br>Four 50 kcal packs mini M&M's	Face-to-face experiment Observe M&M's and complete a questionnaire on snack perceptions	None	Perception (diet food characteris-tics and energy content)[14]	1. Participants perceived that mini M&M's in smaller multipacks contain significantly more energy (144 kcal/75%) than regular-sized M&M's in a larger pack, $p<0.01$.<br>No difference in perception of diet food characteristic was found between package size conditions, $p>0.1$.<br>2. Participants perceived mini M&Ms in smaller multipacks to be significantly more like diet food than regular-sized M&M's in larger packs, $p<0.01$.
Van Kleef, 2014 Study 2 (Netherland)<br>To examine whether the unit size of food impacts intake as it influences perceptions of impulsiveness and appropriateness.	124 university students (75 female)	Quasi	Mars chocolate bars; in original manufacturer package.<br>Between-subjects design; participants randomly assigned to package conditions.<br><br>One 51g pack (228 kcal in total)<br>Five 10 g packs (224 kcal in total)	Face-to-face experiment; Participants instructed to finish provided chocolate in classroom, followed by a questionnaire on snack perception	None	Perception (perceived energy intake)	1. Participants overestimated their energy intake more significantly when eating from smaller multipacks (43% more) than a larger pack (4% more), $p<0.01$.<br>2. Participants perceived that finishing provided chocolates in smaller multipacks as significantly less appropriate, more excessive and more impulsive (all $ps<0.05$), resulted in lower expected satiation and satiety ($p<0.01$) than finishing that in a larger pack.<br>3. No interacting effect of chocolate healthiness was observed between different package size conditions, $p>0.22$.

1. Quasi: quasi-experimental studies.
2. RCT: randomised-controlled trials.
3. Appearance self-esteem (ASE): the self-worth a person derives from his or her body-image and weight.
4. Self-regulatory concern activated group: participants were instructed to complete a body image satisfaction scale and dieting scale, and report their weight before the study; Self-regulatory concern inactivated group (control group): participants participated in an unrelated study before the study.
5. Diet-consciousness activated group: participants were instructed to complete a body image questionnaire, self-reported height, and weight before the study to manipulate diet-consciousness; Diet-consciousness inactivated group (control group): the same questionnaire was given to participants but after food exposure.

[6.] Measured diet-consciousness: participants were categorised into high and low diet-consciousness groups by a dietary restraint scale questionnaire.
[7.] Diet-consciousness was activated in all participants, participants were instructed to complete a body image questionnaire and report height and weight before the study.
[8.] Food focus: participants were instructed to evaluate the M&Ms while eating.
[9.] Perceived norm of appropriate intake: the perception of appropriate serving size (the appropriate amount of food to consume per eating occasion).
[10.] Self-control conflict: the offer of tempting food to a consumer who is occupied with restraining food intake (i.e., commitment to a health goal) sparks a self-control threat that evokes feelings of conflict. This conflict experience operates as an alarm that signals the need to restrain food intake. Failing to evoke this conflict leads to a failure to exert self-control, which then contributes to overconsumption.
[11.] Dietary restraint status: linked with individual's perceived ability to estimate energy in this study. Restrained eaters perceived that they have strong ability to make energy estimation. Unrestrained eaters perceived that they lack ability to make energy estimation.
[12.] Diet prime conditions: (1) Food-focus: participants were instructed to 'think about the sensory experience of enjoying M&Ms' such as the texture and taste; (2) Non-food focus: participants were instructed to 'think about the M&Ms as 'non-food objects'; (3.) Control condition (no food focus): participants were instructed to 'think about anything you would like to think'.
[13.] Perception of impulsiveness: participants were instructed to consider the amount of chocolate they consumed and answered five questions on self-perceived impulsiveness (for example, '... am self-indulgent', '... cannot resist the temptation of chocolate').
[14.] Diet food characteristics: measured by 7-point scale on the extent to which they disagree/agree with the statement 'Overall, the M&Ms in their packages seemed like diet foods'.

Table S4: Supplementary information of intervention studies comparing packaged versus unpackaged energy-dense, nutrient-poor containing same total serving size

First author, Year of publication, Country	Study sample	Study type	Experimental method	Setting	Potential moderators or mediators	Outcome measures	Results
Argo, 2012 Study 1 (Canada)<br>To examine whether people low in appearance self-esteem are particularly sensitive to external control properties.	76 female under-graduate students	Quasi[1]	Gumdrops; the total serving size was 85 gumdrops.<br>Between-subjects design<br><br>Loose gumdrops in s bowl (poured from a new large package at the beginning of experiment)<br>Five 17 g small packs in a bowl	Face-to-face experiment<br>Complete a survey on gumdrops and appearance self-esteem (ASE) while snacking in laboratory setting	Appearance self-esteem (ASE)	Consumption	1. Participants consumed significantly more from snacks that were packaged than that were loose, p<0.05. This was fully contributed by those with low-ASE.<br>2. Participants with low ASE consumed significantly more (22 g/ 129%) from snacks that were packaged than that were loose (Mean packaged=39 g vs Mean loose=17 g), p<0.01.<br>2. No effect of the presence of package on consumption was found among participants with high-ASE (Mean packaged = 24 g vs Mean loose = 23g), p>0.6.
Argo, 2012 Study 4 (Canada)<br>To examine whether people low in appearance self-esteem are particularly sensitive to external control properties.	297 female under-graduate students	Quasi	Candy-coated chocolates; energy information (high vs low vs absent) shown on package; the total serving size was 88 chocolates.<br>Between-subjects design.<br><br>A bowl of loose chocolates<br>Eight small packs (11 chocolates per pack)	Face-to-face experiment<br>Complete a survey on snacks (chocolates) while snacking in laboratory setting	ASE<br>Energy information labelling	Consumption	1. Participants consumed significantly more when snacks were in small packages compared to when snacks were loose, which was fully contributed by those with low-ASE (28 g/350% more from packaged than loose snacks).<br>2. No effect of the presence of package on consumption was found among participants with high-ASE.<br>3. When package was present, participants with low ASE consumed more when they were informed that the energy content of packaged chocolates was low, compared with when energy content was high or when there was no energy content information (Mean low=70 g vs Mean high=24 g vs Mean absent=14 g), p<0.05.<br>4. ASE and energy information labelling were not moderators when chocolates were loose.
Argo, 2012 Study 5 (Canada)<br>To examine whether people low in appearance self-esteem are particularly	105 female under-graduate students	Quasi	Candy-coated chocolates; total serving size was 88 chocolates.<br>Between-subjects design.<br><br>A bowl of loose chocolates<br>Eight small packs (11 chocolates per package)	Face-to-face experiment<br>Complete a survey on provided chocolates while snacking in laboratory setting	ASE<br>Cognitive load (memorising numbers)[3]	Consumption	1. Participants with low-ASE consumed significant more (17 g/81%) from snacks that were packaged than that were loose (Mean packaged=38 g vs Mean loose=21 g), p<0.05.<br>2. Participants in the low cognitive load condition consumed significantly more (14 g/74%) from snacks that were packages than from snacks that were loose (Mean packaged=33 g vs Mean loose=19 g), p<0.01.

sensitive to external control properties.							3. No effect of the presence of small package on consumption was found in participants with high-ASE or when participants' cognitive load was high.
Chance, 2014<br>Study 2<br>(USA)<br>To test the effect of three serving size manipulations on reducing candy consumption in office kitchens.	Office micro kitchens of a technology company	Quasi (interrupted-times-series)	M&M's<br>Consumption at each occasion was observed by research assistants at baseline (when bulk containers present), and during intervention (when small fun packs present for 4 weeks).<br><br>Loose M&M's in a bulk container<br>M&M's in small fun packs	Free-living environment<br>M&Ms were placed in office kitchens for consumption.	None	Consumption	Participants consumed significantly less (178 kcal/58%) on each occasion when snacks were in small packages (fun packs) than when snacks were loosely in the bulk container (Mean small=130 kcal vs Mean large=308 kcal), $p<0.001$.
Knowles, 2020<br>Study 1<br>(UK)<br>To explore the proximity effect that whether distance between consumer and snacks reduces intake.	80 university students (68 females<br>Mean age 21 years<br>Mean BMI 24.9kg/m2	RCT[2]	Brownies (around 10 g each). The total serving size was 20 brownies.<br>Between-subjects design; participants randomly assigned to one of the package conditions<br><br>Unwrapped in a transparent bowl<br>Wrapped individually in plastic film in a transparent bowl	Face-to-face experiment<br>Complete a survey while snacking in laboratory setting for 10 mins.	Perceived effort[4]<br>Visual salience[5]	Consumption	. Participants consumed significantly less from snacks that were packaged than that were unwrapped in a bowl, $p<0.05$.<br>2. The perceived effort was a moderator. Unwrapped snacks required less perceived effort to attain than wrapped snacks, $p<0.01$.<br>3. The visual salience was a moderator. Unwrapped snacks had a higher visual salience than wrapped snacks, $p<0.01$.
Cheema, 2008<br>Study 1<br>(USA)<br>To explore the effect of partitioning on consumption in real life setting.	22 under-graduate students (all females)	Quasi	Chocolates; re-packed in a sealed box; total serving size was 6 pieces of chocolates.<br>Between-subjects design.<br><br>Unwrapped in a box<br>Wrapped individually foil in a box	Free-living environment<br>Participants consumed chocolates in real life and were instructed to finish all chocolates in one week	Self-regulatory concern (aversion to overconsume)	Rate of consumption	1. All participants were required to finish provided chocolates in a week. Participants consumed wrapped chocolates significantly more slowly than that were unwrapped (consumed 45 out of 66 pieces in total if chocolates were wrapped vs 60 out of 66 in total if unwrapped, in first two days).<br>This effect was fully contributed by participants with greater self-regulatory concerns. They spent 4.25 more days to finish wrapped chocolates (Mean unwrapped 1.25 vs Mean wrapped 5.5 days), $p<0.01$.<br>2. No significant effect was found among participants with no self-regulatory concern.

Cheema, 2008 Study 4 (USA) To examine the effect of different partitions (package colour) on consumption.	54 university students	RCT	Cookies; re-packed in container or wax paper; total serving size was 20 cookies. Between subjects design; participants randomly assigned to one of the three package conditions.<br><br>Unwrapped<br>Wrapped individually in white wax paper<br>Wrapped individually in different colour	Face-to-face experiment Consumption was observed uninterruptedly for 140 mins in laboratory setting (while completing an unrelated experiment)	Package colour	Rate of consumption	1. Participants consumed cookies that were individually wrapped in coloured packages significantly more slowly (140 mins) than those that were wrapped in white packages (99 mins) or those that were unwrapped (94 mins), $p<0.001$.<br>2. Participants with cookies wrapped in white packages had the same consumption rate as participants with unwrapped cookies, $p=0.9$.<br>3. Seventeen of 20 participants finished cookies that were individually wrapped in coloured packages during the experiment; all 20 participants finished cookies were individually wrapped in white packages or unwrapped.

[1.] Quasi: quasi-experimental studies.

[2.] RCT: randomised-controlled trials.

[3.] Cognitive load: participants were given a memory task (memorising numbers) at the start. Participants in low cognitive load condition were required to memorise a two-digit number whereas those in high cognitive load condition were required to remember an eight-digit number.

[4.] Perceived effort: the required effort to attain the provided snack.

[5.] Visual salience: the subjective perception of attractive properties of the provided snack.

Table S5: Risk of bias assessment using the Joanna Briggs Institute Checklist for randomised controlled trails (n=26)

**References**	**1. True randomi-sation used for assignment of participants to treatment groups**	**2. Allocation to treatment groups concealed**	**3. Treatment groups similar at baseline**	**4. Participants blind to treatment assignment**	**5. Those delivering treatment blind to treatment assignment**	**6. Outcome assessors blind to treatment assignment**	**7. Treatment groups treated identically other than the intervention of interest**	**8. Follow up complete and if not, differences between groups in terms of their follow up were adequately described and analysed**	**9. Participants analysed in the groups to which they were randomised**	**10. Outcomes measured in the same way for treatment groups**	**11. Outcomes measured in a reliable way**	**12. Appropriate statistical analysis used**	**13. Appropriate trial design, and deviations from the standard design accounted for in the conduct and analysis**	**Risk of bias[a]**
Aerts 2017 Study 1	U	N	Y	Y	N/A	N/A	Y	Y	Y	Y	Y	Y	Y	Low
Aerts 2017 Study 2	U	U	Y	Y	N/A	N/A	Y	Y	Y	Y	Y	Y	Y	Low
Cheema 2008 Study 4	U	U	U	Y	N/A	N	Y	Y	Y	Y	Y	U	Y	High
Clarke 2020	Y	Y	Y	U	N/A	N/A	Y	Y	Y	Y	Y	Y	Y	Low
Codeling 2020	Y	U	Y	Y	N/A	N/A	Y	Y	Y	Y	Y	Y	N	Low
Do Vale 2008 Study 2	U	U	U	Y	N	N/A	Y	Y	N	Y	Y	Y	Y	High
Haire 2014	Y	U	Y	Y	U	N/A	Y	U	N	Y	Y	Y	Y	High

John 2017 Study 1	U	Y	U	Y	N/A	N/A	Y	Y	Y	Y	Y	Y	Y	Low
John 2017 Study 2	U	Y	U	Y	N/A	N/A	Y	Y	Y	Y	Y	Y	Y	Low
John 2017 Study 3a	U	Y	U	U	N/A	N/A	Y	Y	Y	Y	Y	Y	Y	High
Knowles 2020 Study 1	Y	U	Y	Y	N/A	N/A	Y	Y	N	Y	Y	N	Y	Low
Mantzari 2017	Y	U	Y	Y	N/A	N/A	Y	Y	Y	Y	Y	Y	N	Low
Mantzari 2020	Y	N	Y	Y	N/A	N/A	Y	Y	Y	Y	Y	Y	N	Low
Marchiori 2012	U	U	Y	Y	N/A	N/A	Y	Y	Y	Y	Y	Y	Y	Low
Raynor 2007	U	U	Y	Y	U	U	Y	Y	N/A	Y	Y	Y	Y	High
Rolls 2004	U	U	Y	N	U	U	Y	Y	Y	Y	Y	Y	U	High
Roose 2017 Study 2	U	U	U	Y	N/A	N/A	Y	Y	Y	Y	Y	Y	Y	High
Stroebele 2009	U	U	Y	U	U	U	Y	Y	N/A	Y	Y	Y	N	High
VanKleef Study 3	U	U	Y	Y	N/A	N/A	Y	Y	N	Y	Y	Y	Y	High
Versluis 2015 Study 1	U	U	U	Y	N/A	N/A	Y	Y	N	Y	Y	Y	Y	High
Versluis 2015 Study 2	U	U	U	Y	N/A	N/A	Y	Y	N	Y	Y	Y	Y	High

Versluis 2016 Study 1	U	U	Y	Y	N/A	N/A	Y	Y	N/A	Y	Y	Y	Y	Low
Versluis 2016 Study 2	U	U	Y	Y	N/A	N/A	Y	Y	Y	Y	Y	Y	Y	Low
Wansink 2001	U	U	U	N	N/A	N/A	Y	Y	Y	Y	Y	Y	Y	High
Wansink 2005	U	U	Y	N	N/A	N/A	Y	Y	Y	U	Y	Y	Y	High
Wansink 2011	U	U	Y	Y	N/A	N/A	Y	Y	N	Y	Y	Y	Y	High

[a] Risk of bias assessed as high if three or more criteria are assessed as N or U, otherwise assessed as Low.

Table S6: Risk of bias assessment using the Joanna Briggs Institute Checklist for quasi-experimental studies (n=20)

**References**	**1. The 'cause' and the 'effect' are clear (i.e. there is no confusion about which variable comes first)**	**2. Participants included in any comparisons similar**	**3. Participants included in any comparisons receiving similar treatment/care, other than the exposure or intervention of interest**	**4. There was a control group**	**5. Multiple measurements of the outcome both pre and post the intervention/ exposure**	**6. Follow up complete and if not, differences between groups in terms of their follow up adequately described and analysed**	**7. Outcomes of participants included in any comparisons measured in the same way**	**8. Outcomes measured in a reliable way?**	**9. Appropriate statistical analysis used?**	**Risk of bias[a]**
Argo 2012 Study 1	Y	U	Y	Y	Y	Y	Y	Y	Y	Low
Argo 2012 Study 2	Y	U	Y	Y	Y	Y	Y	Y	Y	Low
Argo 2012 Study 4	Y	U	Y	Y	Y	Y	Y	Y	Y	Low
Argo 2012 Study 5	Y	U	Y	N	Y	Y	Y	Y	Y	Low

Bui 2017 Study 1	Y	U	Y	Y	N/A	Y	Y	N	Y	Low
Bui 2017 Study 2	Y	U	Y	N	N/A	Y	Y	N	Y	High
Bui 2017 Study 3	Y	U	Y	Y	Y	Y	Y	Y	Y	Low
Chance 2014	Y	U	Y	N	Y	N/A	Y	Y	U	High
Cheema 2008 Study 1	Y	U	Y	Y	Y	N	Y	N	Y	High
Holden 2015 Study 1	Y	U	Y	Y	Y	Y	Y	Y	Y	Low
Holden 2015 Study 2	Y	U	Y	N	Y	Y	Y	U	Y	High
Huyghe 2013	Y	U	Y	N	N/A	Y	Y	U	Y	High
Kerameas 2015 Study 1	Y	Y	Y	Y	Y	Y	Y	Y	Y	Low
Scott 2008 Study 1	Y	U	Y	Y	N	Y	Y	N	Y	High
Scott 2008 Study 2	Y	U	N	N	Y	Y	Y	Y	Y	High
Scott 2008 Study 3	Y	U	N	N	Y	Y	Y	Y	Y	High
Scott 2008 Study 3 follow-up	Y	U	N	N	N/A	Y	Y	N	U	High
Scott 2008 Study 4	Y	U	N	N	Y	Y	Y	Y	Y	High
Van Kleef 2014 Study 2	Y	U	Y	N	N/A	Y	Y	N	Y	High
Wansink 1996 Study 4	Y	U	Y	N	N/A	Y	Y	N	Y	High

[a] Risk of bias assessed as high if three or more criteria are assessed as N or U, otherwise assessed as Low.

Table S7: Risk of bias assessment using the Joanna Briggs Institute Checklist for qualitative research (n=1)

**References**	**1. Congruity between the stated philosophical perspective and the research methodology**	**2. Congruity between the research methodology and the research question or objectives**	**3. Congruity between the research methodology and the methods used to collect data**	**4. Congruity between the research methodology and the representation and analysis of data**	**5. Congruity between the research methodology and the interpretation of results**	**6. A statement locating the researcher culturally or theoretically**	**7. Influence of the researcher on the research, and vice-versa, addressed**	**8. Participants, and their voices adequately represented**	**9. The research is ethical according to current criteria or, for recent studies, and there is evidence of ethical approval by an appropriate body**	**10. Conclusions drawn in the research report flow from the analysis, or interpretation, of the data**	**Risk of bias[a]**
Mantzari 2018	U	Y	Y	Y	Y	Y	Y	Y	Y	Y	Low

[a] Risk of bias assessed as high if three or more criteria are assessed as N or U, otherwise assessed as Low.
